# Retraction of “Lidocaine has antitumor effect on hepatocellular carcinoma via the circ_DYNC1H1/miR-520a-3p/USP14 axis”

**DOI:** 10.1515/biol-2022-0049

**Published:** 2022-04-21

**Authors:** Hua Liu, Jing Cheng, Heng Xu, Zhenzhen Wan

**Affiliations:** Department of Anesthesiology, Maternal and Child Health Hospital of Hubei Province, Tongji Medical College, Huazhong University of Science and Technology, No. 745 WuLuo Road, Hongshan District, Wuhan City, Hubei Province, China; Department of Anesthesiology, Maternal and Child Health Hospital of Hubei Province, Tongji Medical College, Huazhong University of Science and Technology, Wuhan City, Hubei Province, China

**Retraction of:** Hua Liu, Jing Cheng, Heng Xu and Zhenzhen Wan. Lidocaine has antitumor effect on hepatocellular carcinoma via the circ_DYNC1H1/miR-520a-3p/USP14 axis. Open Life Sciences. Volume 16, Issue 1, doi: 10.1515/biol-2021-0072.

“Lidocaine has antitumor effect on hepatocellular carcinoma via the circ_DYNC1H1/miR-520a-3p/USP14 axis” (https://doi.org/10.1515/biol-2021-0072) has been retracted due to the previously undisclosed conflict of interest between the authors. Authors apologize to the entire scientific community and Editorial team for any issues ensuant from this action.

